# Composition Dependence of Structural and Electronic Properties of Quaternary InGaNBi

**DOI:** 10.1186/s11671-019-2968-0

**Published:** 2019-05-28

**Authors:** Dan Liang, Pengfei Zhu, Lihong Han, Tao Zhang, Yang Li, Shanjun Li, Shumin Wang, Pengfei Lu

**Affiliations:** 1 State Key Laboratory of Information Photonics and Optical Communications, Beijing University of Posts and Telecommunications, Beijing, 100876 China; 2 College of Electrical Engineering and Information Technology, Sichuan University, Chengdu, 610065 China; 3 State Key Laboratory of Functional Materials for Informatics, Shanghai Institute of Microsystem and Information Technology, Chinese Academy of Sciences, Shanghai, 200050 China; 4 Photonics Laboratory, Department of Microtechnology and Nanoscience, Chalmers University of Technology, Gothenburg, 41296 Sweden

**Keywords:** Quaternary, InGaNBi, First-principles, Electronic, Strain

## Abstract

To realize feasible band structure engineering and hence enhanced luminescence efficiency, InGaNBi is an attractive alloy which may be exploited in photonic devices of visible light and mid-infrared. In present study, the structural, electronic properties such as bandgap, spin-orbit splitting energy, and substrate strain of InGaNBi versus In and Bi compositions are studied by using first-principles calculations. The lattice parameters increase almost linearly with increasing In and Bi compositions. By bismuth doping, the quaternary InGaNBi bandgap could cover a wide energy range from 3.273 to 0.651 eV for Bi up to 9.375% and In up to 50%, corresponding to the wavelength range from 0.38-1.9 µm. The calculated spin-orbit splitting energy are about 0.220 eV for 3.125%, 0.360 eV for 6.25%, and 0.600 eV for 9.375% Bi, respectively. We have also shown the strain of InGaNBi on GaN; it indicates that through adjusting In and Bi compositions, InGaNBi can be designed on GaN with an acceptable strain.

## Introduction

In recent years, wurtzite (WZ) *In*_*x*_*Ga*_1−*x*_N alloys and InGaN/GaN quantum wells (QWs) have aroused wide attention due to their large potential for developing solar cells, high-efficiency light emitting diodes (LEDs), and laser diodes (LDs) [1–10]. The commonly used [0001]-oriented *In*_*x*_*Ga*_1−*x*_N/GaN QWs suffer an intense built-in electric field induced by biaxial compressive stress of the *In*_*x*_*Ga*_1−*x*_N layer [11], which gives rise to the decrease in QW emission energy and oscillator strength of electron-hole pairs. Besides, there exists a high-density of geometric defects in *In*_*x*_*Ga*_1−*x*_N alloys, including stacking faults and threading dislocations (TDs) [12]; these TDs have a large correlation with non-radiative recombination centers. Defects, electron leakage, and Auger recombination are the three sources for the efficiency droop of *In*_*x*_*Ga*_1−*x*_N LEDs, of which the Auger recombination is the principal cause [13].

Similarly, for GaAs-based infrared diodes, it has already been proposed that bismuth alloying is an effective method to decrease bandgap (*E*_*g*_) as well as enhance spin-orbit (SO) splitting to achieve the suppression of Auger recombination process [14]. The largest group V element of bismuth reveals attractive effects on physical properties of bismide alloys. The changes in the band structure of bismide alloys have been investigated for different ternary alloy materials both experimentally and theoretically, such as AlNBi [15], GaNBi [16, 17], GaSbBi [18, 19], InPBi [20, 21], and InSbBi [19, 22–24]. The bandgap is modified mainly by the large Bi atom-induced strain at high concentration in InPBi. The incorporation of Bi perturbs the valence bands (VBs) due to the interaction of Bi impurity states with heavy/light hole bands and spin-orbit split off bands [21]. More recently, quaternary bismide alloys (for example, GaAsNBi [25–27], InGaAsBi [28, 29], GaAsPBi [30]) have also garnered extensive attention. The local distortions around P and Bi atoms significantly contribute to the bandgap modification of GaAsPBi. A composition requirement for Ga *As*_1−*x*−*y*_*P*_*y*_*Bi*_*x*_ to achieve lower Auger recombination ratio than GaAs was obtained [30]. Combining bismuth and other III or V atom increases the scope of band structure engineering, including control of bandgap, spin-orbit splitting, conduction (CB) and valence band offsets, and strain [25]. Therefore, it is of significant interest to describe the effect of Bi substitution on the [0001] *In*_*x*_*Ga*_1−*x*_N/GaN, tuning the structural and electronic properties and hence the luminescence efficiency. In present study, using first-principles calculations [31], the structural, electronic properties such as bandgap, spin-orbit splitting energy (*Δ*_*SO*_), and substrate strain of InGaNBi versus In and Bi compositions are studied. Considering the large lattice mismatch and poor quality for In content higher than 55–60% in InGaN sample [32] as well as the low solubility of bismuth in diluted-bismide alloys, the concentrations of In and Bi are controlled up to 50% and 9.375%, respectively. The paper is organized as follows. In the “Methods” section, we present the detailed computational methods. The structural, electronic properties and substrate strain are provided in the “Results and Discussion” section. Finally, a short summary is summarized.

## Methods

Our theoretical calculations are based on the density functional theory (DFT) [31] as implemented in the VASP code [33, 34]. In the calculations of structural properties, the electron-ion and exchange-correlation interactions are treated with the projector augmented wave method (PAW) [35, 36] and the generalized gradient approximation (GGA) of the Perdew-Burke-Ernzerhof (PBE) [37], respectively. The valence-electron configurations for In, Ga, N, and Bi atoms are employed as 4*d*^10^5*s*^2^5*p*^1^, 3*d*^10^4*s*^2^4*p*^1^, 2*s*^2^2*p*^3^, and 5*d*^10^6*s*^2^6*p*^3^, respectively. In order to overcome the underestimation of PBE potential on the bandgap of the electronic properties, we employ the modified Becke-Johnson exchange potential in combination with local density approximation correlation (MBJLDA) [38]. Bismuth has a large spin-orbit coupling (SOC) effect, and therefore, SOC is included in the electronic calculations. In all the computations, the structures are relaxed until the forces on each atom become less than 0.02 eV/Å and maximum energy change is of the order of 10^−4^ eV. A plane-wave cutoff of 450 eV is set to ensure the accuracy of the calculations. A Monkhorst-Pack of 4×4×4*k*-point mesh is adopted in the first Brillouin zone.

## Results and Discussion

### Structural Properties

**Fig. 1:**
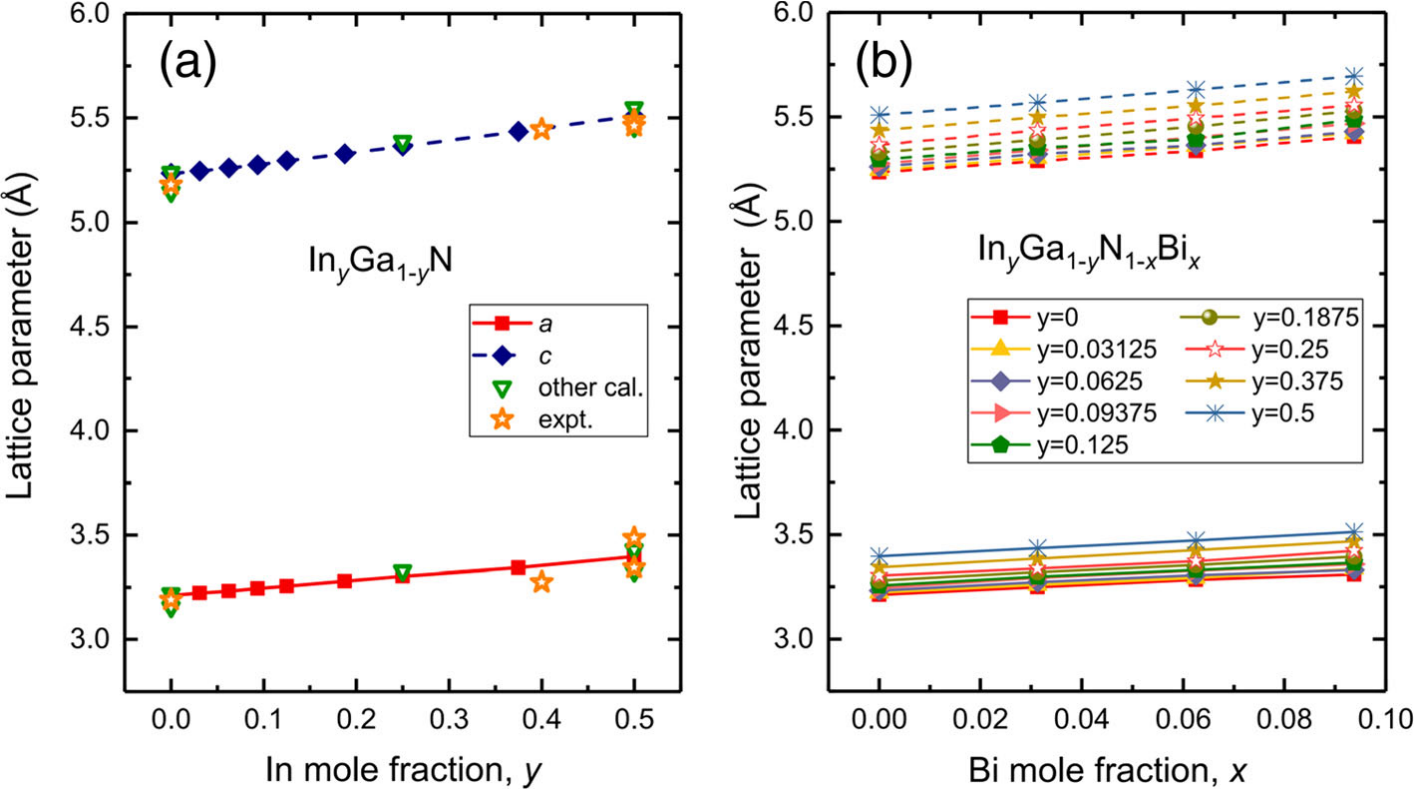
The lattice parameters for **a** ternary alloys *In*_*y*_*Ga*_1−*y*_*N*, with 0≤*y*≤0.5 and **b** quaternary alloys *In*_*y*_*Ga*_1−*y*_*N*_1−*x*_*Bi*_*x*_, with 0≤*x*≤0.09375, 0≤*y*≤0.5. For comparison, we add some other calculations and experimental data from Ref. [39–44] in Fig. 1a. The solid line represents *a* and dashed line is *c*

The supercells consist of 4×2×2 of WZ-GaN primitive cell, including 64 atoms. We investigate 36 compositions of *In*_*y*_*Ga*_1−*y*_*N*_1−*x*_*Bi*_*x*_ with 0≤*x*≤0.09375,0≤*y*≤0.5 based on recent experiments where InGaN sample exhibits large lattice mismatch and poor quality for In content higher than 55–60% [32] as well as the low solubility of bismuth in diluted-bismide alloys. One representative configuration is considered where In and Bi atoms are evenly spread out. We have summarized the calculated lattice parameters of ternary *In*_*y*_*Ga*_1−*y*_N and quaternary *In*_*y*_*Ga*_1−*y*_*N*_1−*x*_*Bi*_*x*_ alloys together with other theoretical and experimental data in Fig. 1. For pristine GaN, the lattice parameters *a*=3.211, *c*=5.235 Å, which are in good agreement with other theoretical calculations *a*=3.155,3.22 Å, *c*=5.144,5.24 Å [39–41] and experimental data 3.19 Å for *a*, 5.19 Å for *c* [42]. The lattice parameters (*a*, *c*) of *In*_*y*_*Ga*_1−*y*_N rise when In composition is increased and show a nearly linear variation, as shown in Fig. 1a. The present calculations predict *a*=3.304 Å, *c*=5.365 Å for *In*_0.25_GaN and *a*=3.397 Å, *c*=5.509 Å for *In*_0.5_GaN, all of which agree well with previous results of *a* = 3.33 Å, *c* = 5.39 Å for *In*_0.25_GaN and *a*=3.43,3.485 Å, *c*=5.55,5.488 Å for *In*_0.5_GaN [39, 40, 43, 44]. In the case of quaternary alloys *In*_*y*_*Ga*_1−*y*_*N*_1−*x*_*Bi*_*x*_, as far as we are concerned, there are no experimental and theoretical values for structural properties. In Fig. 1b, the obtained lattice parameters also increase almost linearly with increasing In and Bi compositions. Because of larger ionic radii of In and Bi than Ga and N atoms, the substitution of In over Ga and Bi over N leads enhanced lattice parameters of InGaNBi.

**Fig. 2:**
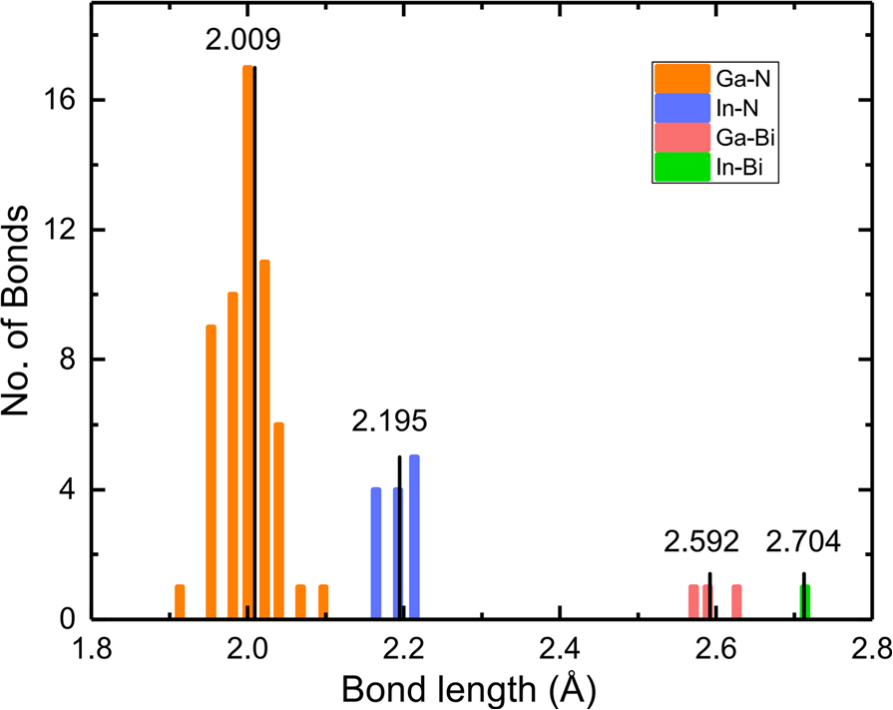
Histogram of bond length in *In*_0.25_*GaNBi*_0.0625_. The values in panel indicate the average lengths of the four types of bond

In and Bi incorporation will break the crystal periodicity and introduce geometrical deformation in a heavily alloyed structure. We choose *In*_0.25_*GaNBi*_0.0625_ as an example for four chemical bonds statistics, as shown in Fig. 2; the average lengths of the Ga-N, In-N, Ga-Bi, and In-Bi bonds are 2.009, 2.195, 2.592, and 2.704 Å, respectively. Note that the Ga-N bond length in pristine bulk GaN is 1.970 Å. The In-N bond length is larger than that of Ga-N, which indicates In atom markedly pushes N atom away. Similarly, the larger bond length of Ga-Bi than Ga-N means Bi atom pushes Ga atom away, finding good consistency with the order of covalent radii of Ga (1.22 Å), In (1.42 Å), N (0.71 Å), and Bi (1.48 Å) [45]. Other configurations display the similar behavior. Lattice deformation and disparity in electronegativity between the host and dopant have considerable effect on electronic and optical properties.

### Electronic Properties

**Fig. 3:**
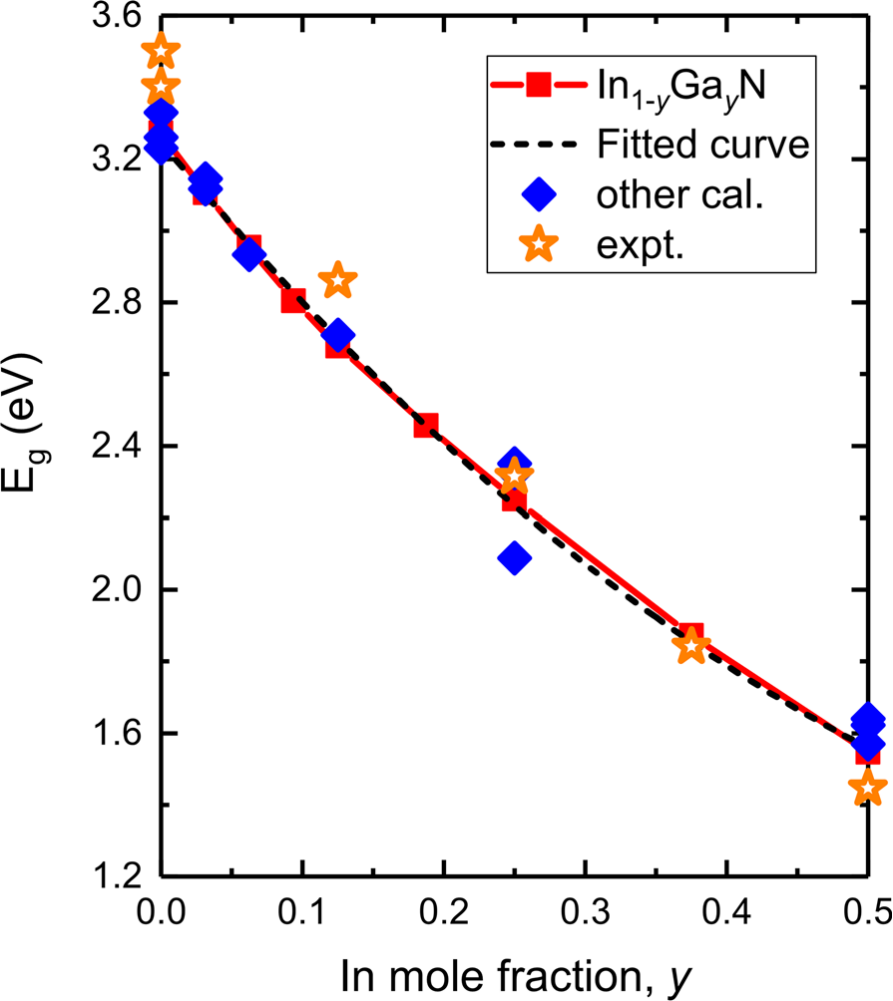
Predicted bandgap energy (*E*_*g*_, red solid line) as a function of In composition in *In*_*y*_*Ga*_1−*y*_*N* as well as a fit to the data (black dashed line). Other theoretical [39, 40, 46] and experimental [47–51] results are also plotted

It has been shown that the functional or correction potentials and SOC effect greatly influence the predicted accuracy of III-V alloy bandgap energy, valence band, and spin-orbit splitting energy. Thus, we validate our results using MBJLDA potential and compare with other theoretical calculations and experiments. Figure 3 is a plot of bandgap energy versus In composition in *In*_*y*_*Ga*_1−*y*_N as well as a fit to the data. Some bandgap values obtained by experiments, theoretical HSE06, mBJ, and LMTO-CPA-MBJ functionals are also plotted. The predicted bandgap of GaN is 3.273 eV, which is in good consistency with present calculations and experiments, 3.33 eV by mBJ [40], 3.261, 3.23 eV by HSE06 [39, 46], and 3.40–3.50 eV by experiments [47–49]. As observed in *In*_*y*_*Ga*_1−*y*_N, our DFT results confirm that *E*_*g*_ values of *In*_*y*_*Ga*_1−*y*_N continuously decrease as *y* is increased from 0 to 50%. *E*_*g*_ smoothly decreases from 3.273 to 1.546 eV. This compares well with those from theoretical (HSE06, mBJ potentials)[39, 40, 46] and experimental results [50, 51].

**Fig. 4:**
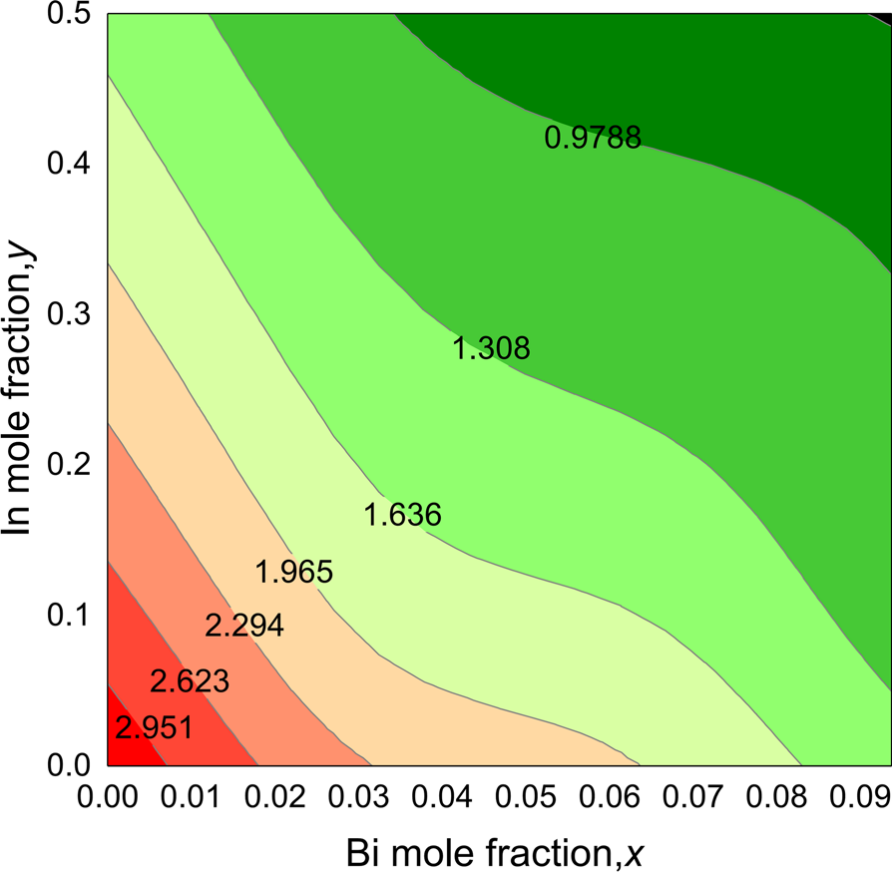
Contour plot of the bandgap values for *In*_*y*_*Ga*_1−*y*_*N*_1−*x*_*Bi*_*x*_ alloys, as a function of Bi(*x*) and In(*y*) compositions

The contour plot for the bandgap of quaternary *In*_*y*_*Ga*_1−*y*_*N*_1−*x*_*Bi*_*x*_ alloys is shown in Fig. 4. The bandgaps of the quaternary alloys display a non-linear trend as a function of composition, which decreases with increasing In and Bi contents. From the results, we find that InGaNBi bandgap could cover a wide energy range from 3.273 to 0.651 eV for Bi up to 9.375% and In up to 50%, corresponding to the wavelength range from 0.38 to 1.9 µm, indicating their potential optoelectronic applications in visible light and mid-infrared scope.

Compared with InGaN, the incorporation of Bi induces a sharper bandgap reduction. But beyond that, a significant increase in *Δ*_*SO*_ is obtained due to the strong SOC effect of bismuth where the advanced interaction between the electron spin and orbital angular momentum decreases the SO band energy. Furthermore, the improved valence-band edge arised from the valence band anti-crossing effect of bismide alloys also largely enhances *Δ*_*SO*_ [28]. Our calculated *Δ*_*SO*_ values are about 0.220 eV for 3.125%, 0.360 eV for 6.25%, and 0.600 eV for 9.375% Bi, respectively, which has an insignificant variation with indium fraction. Previous investigations have demonstrated that different Bi arrangements are of great influence on band structures of bismide alloys, including spin-orbit splitting energy [21, 52]. The present results display that the *In*_0.5_*GaNBi*_0.09375_ bandgap value (0.651 eV) is very close to that of *Δ*_*SO*_ (0.577 eV). Since InGaN sample exhibits large lattice mismatch and poor quality for In content higher than 55–60% [32] as well as the low solubility of bismuth in diluted-bismide alloys, we set the contents of In up to 50% and Bi up to 9.375%. We believe that a higher indium or bismuth content will achieve *Δ*_*SO*_>*E*_*g*_ in quaternary InGaNBi sample to enhance the efficiency of InGaNBi-based LEDs and LDs.

**Fig. 5:**
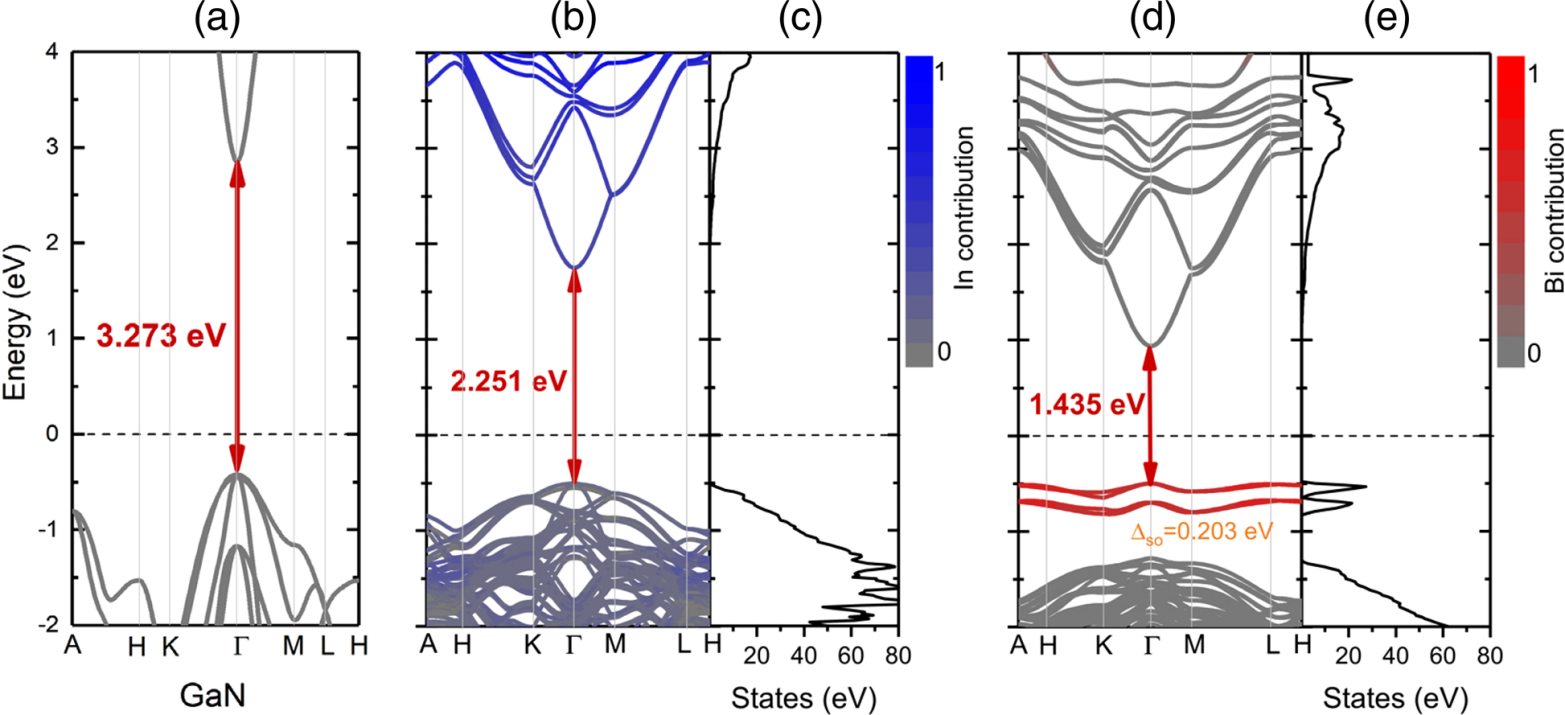
The projected band structures and their corresponding total density of states (TDOS) of **a** GaN, **b**, **c** *In*_0.25_*GaN*, and **d**, **e** *In*_0.25_*GaNBi*_0.03125_. The black dashed line represents the Fermi level, which sets to be zero. The relative contributions of In and Bi are highlighted by color: blue (red) corresponds to the state originating from In (Bi)

The projected band structures and total density of states (TDOS) of pristine GaN, *In*_0.25_GaN, and *In*_0.25_*GaNBi*_0.03125_ alloys are presented in Fig. 5. The contributions of In and Bi are highlighted by color: blue (red) corresponds to the state originating from In (Bi). The In substitution in *In*_0.25_GaN has great influence on both the conduction band and valence band, where the conduction band minimum (CBM) is pushed to lower energies regarding the Fermi level and reflect narrower energy gap. Unlike bismuth that introduces the defect band in the forbidden gap near the Fermi level, the In atoms show a hybridization with the deep level of the VB. For quaternary alloy *In*_0.25_*GaNBi*_0.03125_, it can be clearly seen that the reduction of bandgap results from both upward valence band maximum (VBM) and downward CBM, and CBM changes more significantly compared to *In*_0.25_GaN, which is attributed to larger compressive strain in InGaNBi from the addition of bismuth. The defect level highlighted by red color has a strong interaction with the VB edge, which is derived from the hybridization mainly between Bi and the near Ga atoms. The TDOS in Fig. 5e also reflects the local defect level at −1.0 to −0.5 eV.

### Strain of InGaNBi on GaN

**Fig. 6:**
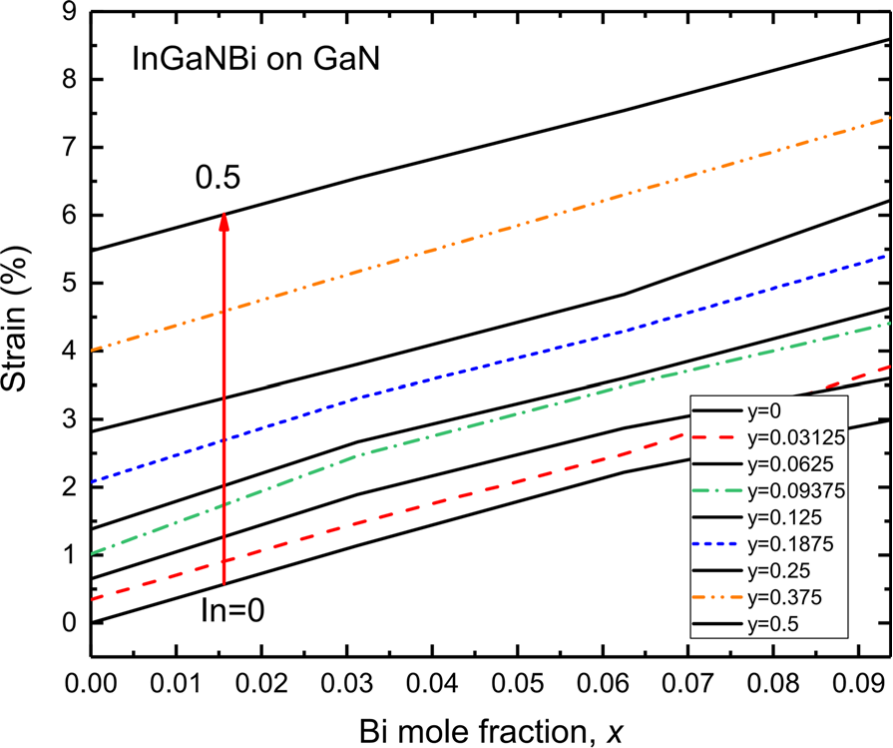
Strain of InGaNBi alloys on GaN substrate at various In (0–0.5) as a function of Bi fraction. Positive values of strain indicate InGaNBi is under compressive strain

The [0001]-oriented *In*_*y*_*Ga*_1−*y*_N/GaN strained quantum wells are widely adopted in current LED and LD devices, in which *In*_*y*_*Ga*_1−*y*_N layers suffer a biaxial compressive stress. Local compositional fluctuations and different covalent radii of In and Ga atoms give rise to the strains in *In*_*y*_*Ga*_1−*y*_N layers [53]. Figure 6 shows the strain of InGaNBi on an GaN substrate. Since indium atom is larger than gallium atom, bismuth atom is larger than nitrogen atom; thus, incorporating In and Bi atoms in InGaNBi induces compressive strain InGaNBi on GaN. It is shown that in the In content of 50% and Bi content of 9.375%, InGaNBi is under high 8.5% compressive strain. For In fraction within 6.25% and Bi fraction within 2.8%, the strain of InGaNBi on GaN is within 1%. That is, through adjusting In and Bi compositions, InGaNBi can be designed on GaN with an acceptable strain.

## Conclusions

The structural, electronic properties and strain of InGaNBi on GaN versus In and Bi compositions are investigated based on density functional theory. The lattice parameters of InGaNBi increase almost linearly with increasing In and Bi compositions. Since In and Bi atoms have the larger atomic radius than that of Ga and N atoms, the In-N and Ga-Bi bond lengths are larger than that of Ga-N. For electronic properties, we have shown the contour plot for the bandgap of quaternary *In*_*y*_*Ga*_1−*y*_*N*_1−*x*_*Bi*_*x*_ alloys. The quaternary alloys bandgap could cover a wide energy range from 3.273 to 0.651 eV for Bi up to 9.375% and In up to 50%, corresponding to the wavelength range from 0.38 to 1.9 µm. The calculated *Δ*_*SO*_ values are about 0.220 eV for 3.125%, 0.360 eV for 6.25%, and 0.600 eV for 9.375% Bi, respectively, which has an insignificant variation with indium fraction. We believe that a higher indium or bismuth composition will achieve *Δ*_*SO*_>*E*_*g*_ in quaternary InGaNBi sample to enhance the efficiency of InGaNBi-based LEDs and LDs. The band structure analyses show the indium has great influence on both CB and VB, and bismuth has a strong interaction with the VB edge. Finally, we investigate the strain of InGaNBi on GaN. Through adjusting In and Bi compositions, InGaNBi can be designed on GaN with an acceptable strain.
